# Subjective judgements – no more, no less? A response to Malterud, Bjelland and Elvbakken

**DOI:** 10.1186/s12961-018-0386-x

**Published:** 2018-11-20

**Authors:** Atle Fretheim

**Affiliations:** Norwegian Institute of Public Health, Oslo, Norway

**Keywords:** Evidence-based medicine, Systematic reviews, Qualitative methods

## Abstract

In 2016, three researchers published a report where they concluded that systematic reviews from my institution, the Norwegian Knowledge Centre for the Health Services, are of little use for health policy decision-making. Based on their reading of the conclusion section in 14 reports, they argue that our systematic reviews are not useful due to their lack of clear and conclusive findings. I have reviewed the same documents and I beg to differ. Unfortunately, the description of their methodological approach is both sparse and difficult to grasp, making it very hard to understand how they arrived at their conclusions.

**Electronic supplementary material:**

The online version of this article (10.1186/s12961-018-0386-x) contains supplementary material, which is available to authorized users.

The Norwegian Knowledge Centre, like many other organisations around the world, produces systematic reviews to support evidence-informed decisions in the health services and in health policy. At the Centre (now part of the Norwegian Institute of Public Health) we prepare systematic reviews for a range of commissioners, including government bodies, professional groups, and patient organisations. Systematic reviews are generally regarded as more reliable than single studies because they provide a more comprehensive representation of the available evidence [1]. In our reports, we classify the certainty of the evidence as high, moderate, low and very low, using the GRADE approach [2].

In their case study from Norway, published in *Health Research Policy and Systems*, Malterud, Bjelland and Elvbakken conclude that the systematic reviews prepared by my institution are inappropriate for health policy decision-making [3]. Their argument is simple, namely that the reports we produce do not provide clear conclusions, and are therefore not useful.

They based their analysis on the conclusions from a “*purposive subsample*” of 14 publications from 2012. In their assessment, these reports “*advised major caution about their conclusions because of the quality or relevance of the underlying documentation*”. They further elaborate that “[o]*nly* *one of these 14 SRs from 2012 (dealing with interventions to prevent use of tobacco, alcohol and drugs among children and adolescents (07-2012)) concluded that extensive and high quality documentation had been identified. While the report identified a range of effective interventions, the language of caution again appeared (e.g. ‘possibly effective’, ‘likely not effective’). Another related SR, dealing with interventions regarding nutrition, physical activity, obesity and sexual health in children and adolescents (06-2012), reported that substantial documentation allowed the authors to draw some conclusions. Nevertheless, they expressed some reservations about the broad scope of the documentation, which meant that the recommended interventions were at a rather general level. For the remaining 12 SRs, the authors’ conclusions are characterized in every case by an overarching caution*”.

Leaving aside the argument that demonstrating uncertainty may be useful in itself, how did Malterud et al. [3] go about assessing the degree of caution expressed by my colleagues? Unfortunately, the description of their methodological approach is both sparse and difficult to grasp:

“*Drawing on perspectives from the rhetorics of health and medicine, we assessed the persuasive power of the conclusions mediated by the language used, especially with regard to terms indicating positions of certainty or reluctance. This process was conducted by systematic negotiation between the authors in pursuit of consensus*”.

Thanks to open peer review, I know that I am not the only one struggling with understanding this. Reviewer 1 wrote: “*I finished the methods not entirely sure I know how you carried out the study, it’s unclear and lacking in detail about the ‘how we did it’ information. The casual reader who will glance through the methods will be even more confused*” [4].

In an attempt to replicate the study, I reviewed the same documents, i.e. the conclusion section from the 14 reports published in 2012 (Additional file 1).

In our reports, review authors generally follow a principle whereby qualifying words such as 'probably' and 'may' are used to signal moderate and low certainty evidence, respectively, while no such modifiers are used if the certainty of the evidence is considered high [5]. This informed my assessment of the clarity of the conclusions.

In my judgement, 4 of the 14 reports included findings without major reservations about uncertainty, e.g. “*Comprehensive school-based interventions to prevent the use of alcohol and marijuana are effective in preventing the use of both alcohol and marijuana among 10-15 year olds*” and “*Health education that targets smoking pregnant women probably helps them to stop smoking*”.

Of the remaining 10 reports, 6 included findings of low certainty, e.g. “*support and follow-up interventions such as education, exercise and vocational rehabilitation may have beneficial effects on health and health related outcomes*”.

In 3 reports, the findings were all of very low certainty, and for 1 report, no evidence was found at all.

My assessment seems to differ from that of Malterud et al. [3]. This is intriguing and worth exploring, but is not possible without a better understanding of their method.

Malterud et al. [3] seem to argue that systematic reviews are less suited for policy processes than for clinical questions: “*A typical example would be whether medication X is a better treatment than medication Y for patients suffering from a single and well-defined disease*”. This may be true, but it is certainly not a given that systematic reviews with a narrow clinical focus provide definitive answers. Interestingly, in the only such systematic review we produced in 2012 (excluded from Malterud et al.’s analysis) we concluded that “[t]*he results are associated with uncertainty as they are based on the efficacy data of a sub-population analysis from only one clinical trial*” [6].

Like Malterud et al., I “*did not include a systematic investigation of uptake and policy consequences*”, and I would certainly agree that proof of the usefulness of systematic reviews lies in their actual use. Rather than discarding them, I would propose to develop methods to increase and improve their use in decision-making processes, e.g. through better formats for conveying results to policy-makers [7].

Therefore, transparent methods are necessary in the evaluation of the usefulness of systematic reviews, as well as of new approaches to improve their usefulness.

## Additional file

Additional file 1: Conclusion sections from the 14 included systematic reviews. (DOCX 34 kb)
