# Supplementary material for: Subjective judgements – no more, no less? A response to Malterud, Bjelland and Elvbakken
Source: Health Res Policy Syst. 2018 Nov 20;16:111. doi: 10.1186/s12961-018-0386-x (PMC6245815; doi:10.1186/s12961-018-0386-x)
Supplement: Supplementary file 1 — Conclusion sections from the 14 included systematic reviews. (DOCX 34 kb) [file 12961_2018_386_MOESM1_ESM.docx]

**Additional file**

**Fretheim A. Subjective judgements – no more, no less? A response to Malterud, Bjelland and Elvbakken.**

The following text forms the basis of Malterud, Bjelland and Elvbakken’s conclusions in their paper *Evidence-based medicine – an appropriate tool for evidence-based health policy? A case study from Norway* (Malterud et al. Health Research Policy and Systems [2016] 14:15):

[**List size and quality of care among GPs within the The Regular General Practitioner Scheme**](http://www.kunnskapssenteret.no/en/publications/list-size-and-quality-of-care-among-gps-within-the-the-regular-general-practitioner-scheme?vis=sammendrag)

**Conclusion**

We did not identify any studies assessing the effect of GPs' list size on the quality of health care services. Thus, we cannot draw any conclusions on whether short or long patient lists are effective in terms of the quality of services provided by GPs.

We found 91 studies that, although they did not evaluate effect, examined the association between GPs' list size and the quality of primary care physician services. We think it is useful to have an overview of available studies. We have therefore described the studies and their main results. We have also elucidated that, among the studies, there is a great variation regarding aims, methods used, and study settings. None of the studies examined what the optimal list size would be in relation to quality of the primary care physician services.

# [The effects of sexual therapy interventions for sexual problems](http://www.kunnskapssenteret.no/en/publications/the-effects-of-sexual-therapy-interventions-for-sexual-problems?vis=sammendrag)

**Conclusion**

Overall, sexual therapeutic interventions are effective for people with sexual problems. This is shown for both a broad spectrum of populations and sexual problems. Self help, as information in written or audio/visual material (bibliotherapy) can be effective in the treatment of sexual problems. The results, however, must be interpreted with caution as the study groups were small, there are limitations in methodological quality and large variations in what is defined and presented as sexual therapeutic interventions. We can not rule out publication bias.

# [Interventions for Tobacco Control in Low- and Middle-income countries: Evidence from Randomised and Quasi-randomised Studies](http://www.kunnskapssenteret.no/en/publications/interventions-for-tobacco-control-in-low-and-middle-income-countries-evidence-from-randomised-and-quasi-randomised-studies?vis=sammendrag)

Conclusion

In low- and middle income countries, nicotine replacement therapy and buproprion may help smokers to stop smoking and probably reduces smoking rates. Health education that targets smoking pregnant women probably helps them to stop smoking, and may result in one or more quit attempts or a reduction in the amount of smoking. We are uncertain of the effect of health education at the primary care or community level on smoking cessation, but this may decrease overall smoking rates.

School- based interventions probably prevent progression to regular smoking among experimenters or non smokers. These interventions may reduce overall smoking rates and improve life skills. School based interventions probably improve knowledge, attitudes and beliefs about the effects of tobacco smoking. We are uncertain if school-based interventions prevent experimentation with cigarettes.

However the evidence base is not very strong as most of the included studies were small, implemented over short periods and at times addressed different questions. There is a need for more rigorous studies conducted in LMICs, perhaps with a particular focus on delivery strategies of therapies that have been successful in high income settings. Some interventions such as those targeting the supply of tobacco, enforcing bans on tobacco advertising or raising taxes require further evaluation, especially in LMIC where the legislation and enforcement of tobacco control varies widely.

[**Effects of organisational interventions for mental health services**](http://www.kunnskapssenteret.no/en/publications/effects-of-organisational-interventions-for-mental-health-services?vis=sammendrag)

Conclusion

Of the identified organizational interventions, intensive case management compared with standard therapy appears to be the intervention with positive effects on more outcomes. The quality of the evidence for these results was low. Overall, the scientific evidence for the effect of organisational interventions is generally of low and very low quality. The main part of the studies was of an earlier date. The scientific evidence appears to be a rather inadequate basis for decisions on future organisation of mental health care.

[**The effectiveness of health promotion and preventive interventions on nutrition, physical activity, obesity, and sexual health in children and adolescents**](http://www.kunnskapssenteret.no/en/publications/the-effectiveness-of-health-promotion-and-preventive-interventions-on-nutrition-physical-activity-obesity-and-sexual-health-in-children-and-adolescents?vis=sammendrag)

Conclusion

The six included systematic reviews showed that:

- School-based initiatives to promote a healthy diet may possibly lead to higher intakes of fruits and vegetables in children and adolescents.
- The documentation was too limited to draw any conclusions about the effectiveness of school-based interventions to promote physical activity in children and adolescents.
- Preventive interventions that focus on physical activity and nutritional education to prevent obesity among 0-5 year-olds may possibly not lead to less obesity.
- The documentation was too limited to draw any conclusions about the effectiveness of preventive interventions that focus on increasing physical activity and healthy eating to prevent obesity in children and adolescents aged 6-18 years.
- Abstinence-only interventions may possibly not be effective in preventing sexually transmitted infections or pregnancy among children and adolescents.
- Abstinence-plus interventions may possibly not be effective in preventing sexually transmitted infections or pregnancy among children and adolescents.

Future research should focus on the long-term effectiveness of interventions with follow-up at least half a year after the program has been completed. There is also a need for systematic reviews that deal with the effectiveness of health promotion for social health in children and adolescents, and studies on the effectiveness of non-school based interventions to prevent obesity and physical inactivity.

[**The effectiveness of primary interventions to prevent the use of tobacco, alcohol and other drugs among children and adolescents**](http://www.kunnskapssenteret.no/en/publications/the-effectiveness-of-primary-interventions-to-prevent-the-use-of-tobacco-alcohol-and-other-drugs-among-children-and-adolescents?vis=sammendrag)

Conclusion

The ten included systematic reviews showed that:

• Comprehensive school-based interventions to prevent the use of alcohol and marijuana are effective in preventing the use of both alcohol and marijuana among 10-15 year olds.

• School-based interventions, which emphasize skills, to prevent the use of illegal drugs may be effective in preventing the use of hard drugs among children and adolescents.

• The following interventions to prevent smoking may possibly be effective: i) mass-media campaigns, ii) school-based interventions that emphasize social skills, iii) parent-based interventions for children aged 5-11 years.

• The following prevention interventions may possibly be effective: i) school-based interventions against illegal drugs which emphasize knowledge to prevent use of marijuana, ii) parent-based interventions to prevent the use of tobacco, alcohol, and other drugs among adolescents aged 11-14 years, iii) family-based interventions to prevent alcohol abuse among children and adolescents, iv) multi-component interventions to prevent alcohol abuse among children and adolescents.

• School-based interventions, which emphasize knowledge, to prevent the use of alcohol and marijuana may not be effective in preventing alcohol abuse among adolescents aged 10-15 years.

• Family-based interventions to prevent smoking among children and adolescents may not be effective in keeping children and adolescents smoke-free.

• The documentation was too limited to draw any conclusions regarding the effectiveness of a range of other drug prevention interventions.

Future research should focus on the long-term effectiveness of substance abuse prevention interventions with follow-up at least half a year, preferably longer, post project completion. There is also a need for high-quality studies concerning the effectiveness of community-based interventions to prevent smoking, non-school-based interventions to prevent the use of marijuana as well as parenting programs to prevent the use of tobacco, alcohol, and other drugs among children and adolescents.

[**Effect of interventions to ease transitions for children and adolescents with disabilities**](http://www.kunnskapssenteret.no/en/publications/effect-of-interventions-to-ease-transitions-for-children-and-adolescents-with-disabilities?vis=sammendrag)

Conclusion

There is uncertainty about whether interventions to facilitate secondary transitions for youth with disabilities are effective. Drawing on existing knowledge we conclude that there is a need for a systematic review of high methodological quality to address the question of which interventions work for which groups under which conditions. We stress that our conclusions are based on the results of a systematic review of reviews. We cannot say with certainty whether there are primary studies of high quality on interventions to facilitate secondary transitions. We contacted the authors of the included systematic reviews on secondary transitions in order to find out if and when their reviews will be updated, and were informed that updates to draw on studies published after 2004 are planned.

For early transitions there is a need for a systematic review of high quality as we have not identified a systematic review on this issue.

[**Interventions for reducing seclusion and restraint in mental health for adults**](http://www.kunnskapssenteret.no/en/publications/interventions-for-reducing-seclusion-and-restraint-in-mental-health-for-adults?vis=sammendrag)

Conclusion
Joint crisis plans may reduce the number of compulsory admissions in mental health for adults. Systematic evaluation of aggressive behaviour in acute psychiatric wards may also reduce the use of restraint and seclusion. Further research is needed in order to draw more robust conclusions about the effect of interventions intended to reduce coercion in mental health care.

There is a need for more comparative studies in Norway.

[**Effects of support and follow-up interventions for people with severe mental illness**](http://www.kunnskapssenteret.no/en/publications/effects-of-support-and-follow-up-interventions-for-people-with-severe-mental-illness?vis=sammendrag)

Conclusion

Based on the available evidence, support and follow-up interventions such as education, exercise and vocational rehabilitation may have beneficial effects on health and health related outcomes such as readmissions and relapse, and outcomes that may be important for prevention of relapse. We also found improvements in adherence to recommended drug use, patient satisfaction and employments.

There is a need for more studies with larger study populations in order to identify meaningful differences. Also, there is a need for evidence on the effects of interventions such as recreational and leisure activities and supported housing and follow-up measures. Future studies should aim to include mortality, quality of life, knowledge, skills, empowerment, patient satisfaction and costs.

[**Effects of organised follow-up of behaviour that may increase risk of disease in adults**](http://www.kunnskapssenteret.no/en/publications/effects-of-organised-follow-up-of-behaviour-that-may-increase-risk-of-disease-in-adults?vis=sammendrag)

Conclusion

Based on our summary of the findings and assessment of the quality of the documentation, we draw the following conclusions:

 Physical activity

- Referral to a local center and follow-up, and training on one’s own with follow up probably increase physical activity in the intervention period and in the short term (3 months after the intervention period).

Diet and physical activity

- We lack documentation of sufficient quality about interventions to conclude about change of diet and physical activity.

Tobacco

- Self-help materials and follow-up may increase abstinence from smoking during the intervention period.
- Referral to a nurse may increase the number of persons who abstain from smoking 6 months after starting the intervention.

Alcohol

- We did not find studies of interventions to reduce alcohol use that met our inclusion criteria.

Need for further research:

- We still need to study effects of interventions provided in ‘healthy living centres’ – concerning both physical activity, smoking cessation, use of alcohol, and interventions targeting physical activity and diet.
- We wish to emphasize the need for consensus among researchers about methods for measuring physical activity to prevent that this is being done in various different ways. There is also a general need for objective measurement methods that are economically ant ethically justifiable. For smoking cessation, increased use of methods for measuring nicotine-exposure would be desirable. Finally, we want to emphasize the need for measuring long term-effects, i.e. a year or more

[**Effect of long-term mechanical ventilation (LTMV) part 1 – neuromuscular disease or central respiratory failure**](http://www.kunnskapssenteret.no/en/publications/effect-of-long-term-mechanical-ventilation-ltmv-part-1-neuromuscular-disease-or-central-respiratory-failure?vis=sammendrag)

Conclusion

- LTMV may be associated with some degree of life extension and improved quality of life for patients with ALS, at least for patients with good bulbar function. The quality of evidence is low, and it is therefore difficult to draw firm conclusions about the real effect.
- LTMV may be associated with life extension in hypoventilated patients with Duchenne muscle dystrophy, but the quality of the evidence is low. Very low quality evidence from a single randomized controlled study, suggests that it is not beneficial to start LTMV too early.
- For patients with various neuromuscular diagnoses it seems like:
  - Initiation of LTMV may be associated with a decrease in the need for hospital admissions.
  - Invasive LTMV is probably associated with greater risk of complications and hospitalizations than non-invasive LTMV.
  - The quality of evidence is very low, and it is therefore not possible to draw firm conclusions about the real effect.

For some diagnoses, including central respiratory failure, we were not able to identify any research fulfilling our inclusion criteria

[**Effect of long-term mechanical ventilation (LTMV) part 2 – thoracic restrictive disorders or adipositas hypoventilation syndrome**](http://www.kunnskapssenteret.no/en/publications/effect-of-long-term-mechanical-ventilation-ltmv-part-2-thoracic-restrictive-disorders-or-adipositas-hypoventilation-syndrome)

Conclusions

Obesity hypoventilation syndrome (OHS)

- We did not identify studies from which we could estimate association between initialisation of LTMV and patient survival.
- Starting LTMV-treatment may be associated with a decrease in the number of hospital admittances and improvements in some parameters related to quality of life and sleep. However, the quality of the evidence is very low and it is not possible to draw clear conclusions about the effectiveness of LTMV.
- One randomized controlled trial compared CPAP versus BiPAP in a selected group of patients known to respond adequately to CPAP. The results of the trial were too imprecise to conclude whether the two method differed from each other with respect to our primary outcome, i.e. survival, hospitalisation, quality of life, and sleep.

Chest wall disease (CWD)

- LTMV may be associated with increased survival compared to long-term oxygen treatment alone, but the quality of the evidence is low and any conclusion is uncertain.
- LTMV may be associated with fewer hospital admissions and improvements in some parameters related to quality of life and sleep. However, the quality of the evidence is very low and it is not possible to draw any clear conclusions based on the best available evidence.

[**Effect of multidisciplinary, team-based rehabilitation, including education, in rheumatoid arthritis**](http://www.kunnskapssenteret.no/en/publications/effect-of-multidisciplinary-team-based-rehabilitation-including-education-in-rheumatoid-arthritis?vis=sammendrag)

Conclusion

When patient education was delivered as sole intervention, there was a significant improvement in symptoms, self-efficacy and knowledge about RA at oe year follow-up in favour of the intervention group. For other outcomes, patient education showed small or no effects. The results from the studies evaluating the effect of multidisciplinary team-based rehabilitation showed, for HAQ physical function, a significantly improved score in favour of the control group at one year follow-up. At two years follow-up there still was a tendency towards better scores in the control group, but the difference was no longer significant. For other outcomes, multidisciplinary, team-based rehabilitation showed small or no effect.

The documentation about effect of multidisciplinary team-based rehabilitation is insufficient, and all conclusions are based on findings from small studies with unclear or high risk of bias. It is important to emphasize that no evidence of effect is not the same as evidence of no effect. In order to assess the effect of multidisciplinary team-based rehabilitation for patients with RA, it will therefore be a need for more research in this field.

Need for further research

Ideally, the randomized controlled trial is the most appropriate study design to study the effect of different interventions, including rehabilitative interventions. Due to among others organizational causes, it might be difficult to conduct these kinds of studies. An alternative in future studies of rehabilitative interventions can be what is called a "stepped wedge randomised trial design", in which the intervention is sequentially introduced (in clusters) over time periods.

[**Psychological treatments for non-specific chronic pain**](http://www.kunnskapssenteret.no/en/publications/psychological-treatments-for-non-specific-chronic-pain?vis=sammendrag)

Conclusion

This overview of reviews of psychological treatment for patients with non-specific chronic pain showed small to moderate effect on pain, thus new studies may alter our conclusions. Subgroup analyses may facilitate the exploration of data, and confirm results to comparable populations. Decision makers faced with implementing psychological treatment to reduce pain may use this overview to inform these decisions and consider the range of treatments available.

Future research should focus 1) on what kind of specific treatment component in psychological treatment work and 2) to identify client populations that may benefit from that specific treatment. It is difficult to distinguish what element may cause the effect for multidisciplinary treatment. Furthermore, there is also a need to conduct well-designed studies on return-to-work and to identify subpopulations that may benefit from that specific treatment.
